# Chia Oil Supplementation Attenuates Obesity‐Induced Hepatic Steatosis, PVAT Inflammation, and Endothelial Dysfunction in Mice

**DOI:** 10.1002/mnfr.70469

**Published:** 2026-04-17

**Authors:** Agatha de Assis‐Ferreira, Gabrielly Muniz‐Cassuce, Thaís Fonte‐Faria, Thamiris de Souza, Marta Citelli, Lenize Costa Reis Marins de Carvalho, Dayane Teixeira Ognibene, Graziele Freitas de Bem, Angela Castro Resende, Christina Barja‐Fidalgo, Simone Vargas da Silva

**Affiliations:** ^1^ Departamento De Biologia Celular Universidade Do Estado do Rio de Janeiro Rio de Janeiro Brazil; ^2^ Instituto De Nutrição Departamento De Nutrição Básica e Experimental Universidade Do Estado do Rio de Janeiro Rio de Janeiro Brazil; ^3^ Departamento De Farmacologia e Psicobiologia Universidade Do Estado do Rio de Janeiro Rio de Janeiro Brazil

**Keywords:** chia oil, hepatic steatosis, macrophage polarization, perivascular adipose tissue, vascular dysfunction

## Abstract

Obesity promotes metabolic disturbances that contribute to hepatic steatosis, adipose tissue dysfunction, and vascular impairments. Perivascular adipose tissue (PVAT) plays a critical role in vascular homeostasis, yet little is known about plant‐derived omega 3 fatty acids modulate PVAT remodeling during obesity. Here, we investigated whether dietary chia oil supplementation, an abundant source of α‐linolenic acid, attenuates metabolic, inflammatory, and vascular dysfunction in obese mice. Male C57BL/6J mice were fed a chow (C) or high‐fat diet (HF) diet for 14 weeks and a subset of high‐fat‐fed mice received chia oil (1.5% v/v) from weeks 8–14 (HC). Chia oil improved insulin sensitivity, increased adiponectin levels, and reduced leptin and hepatic triglyceride accumulation. Histological and molecular analyses showed decreased macrovesicular steatosis and FABP4 expression beside increased CPT‐1α and PGC‐1α, indicating enhanced lipid oxidation. In mesenteric PVAT, chia oil reduced adipocyte hypertrophy and macrophage infiltration while increasing IL‐10 and CD206 expression. Functionally, chia oil restored acetylcholine‐induced vasodilation, reduced norepinephrine‐mediated vasoconstriction, and increased AMPK phosphorylation. Chia oil supplementation ameliorates key features of obesity‐associated metabolic dysfunction, supporting ALA‐rich oils as promising nutritional strategies against metabolic disease.

## Graphical abstract

We studied whether chia oil, rich in healthy omega‐3 fats, could improve health in obese mice. The oil reduced fat accumulation in the liver, decreased inflammation around blood vessels, and helped blood vessels relax better. These results suggest chia oil may help prevent complications of obesity, like fatty liver and heart disease.

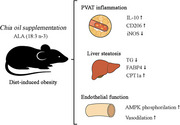

**Abbreviations**
AMP: Activated protein kinase
IL‐10: Interleukin 10
eNOS: Endothelial nitric oxide synthase
iNOS: Inducible nitric oxide synthase
PVAT: Perivascular adipose tissue

## 1 Introduction

Metabolic dysfunction‐associated steatotic liver disease (MASLD) is one of most prevalent metabolic disorders worldwide, affecting approximately one‐third of the global adult population [1]. Closely linked to obesity, dyslipidemia, and insulin resistance, MASLD ranges from simple steatosis to progressive inflammatory and fibrotic stages [2, 3].

Perivascular adipose tissue (PVAT), a specialized adipose depot surrounding blood vessels, has emerged as a key regulator of vascular tone and inflammatory signaling. Under physiological conditions, PVAT exerts vasodilator and anti‐inflammatory effects [4]; however, obesity induces adipocyte hypertrophy, excess lipid deposition, and a pro‐inflammatory secretory profile that contribute to endothelial dysfunction [5, 6].

Omega‐3 polyunsaturated fatty acids, including α‐linolenic acid (ALA), are well recognized for their anti‐inflammatory and metabolic benefits [7, 8, 9, 10]. Although ALA conversion to eicosapentaenoic acid (EPA) and docosahexaenoic acid (DHA) is limited, ALA itself exerts important biological effects and is highly abundant in chia oil. [11, 12, 13, 14]. Previous studies indicate that omega ‐3 fatty acids can improve hepatic lipid metabolism and adipose tissue inflammation [15, 16]. However, the impact of plant‐based ALA‐rich oils on PVAT remodeling, hepatic metabolism, and vascular function in obesity remains poorly defined. The present study aimed to investigate whether dietary chia oil supplementation improves hepatic steatosis, PVAT inflammatory remodeling, and vascular function in obese mice. By integrating metabolic, molecular, histological, and functional analyses, we sought to clarify whether ALA‐rich chia oil could mitigate metabolic dysfunction associated with obesity and MASLD.

## 2 Experimental Section

### 2.1 Diet Composition and Experimental Design

All procedures were approved by the Institutional Committee for Ethics in Animal Use (CEUA‐UERJ, protocol 016/2021) and followed NIH guidelines for the care and use of laboratory animals. Male C57BL6/J mice (3‐weeks‐old) were housed under controlled temperature and 12 h/12 h light–dark cycle. Mice received either a standard chow diet (C: 396 kcal/100 g, 13% energy from fat) or a high‐fat diet (H: 470 kcal/100 g, 45% energy from fat) for 14 weeks. From weeks 8–14, a subset of high‐fat–fed mice received chia oil supplementation (1.5% v/v) (HC group), as described below.

### 2.2 Dosage Information

**FIGURE 1:**
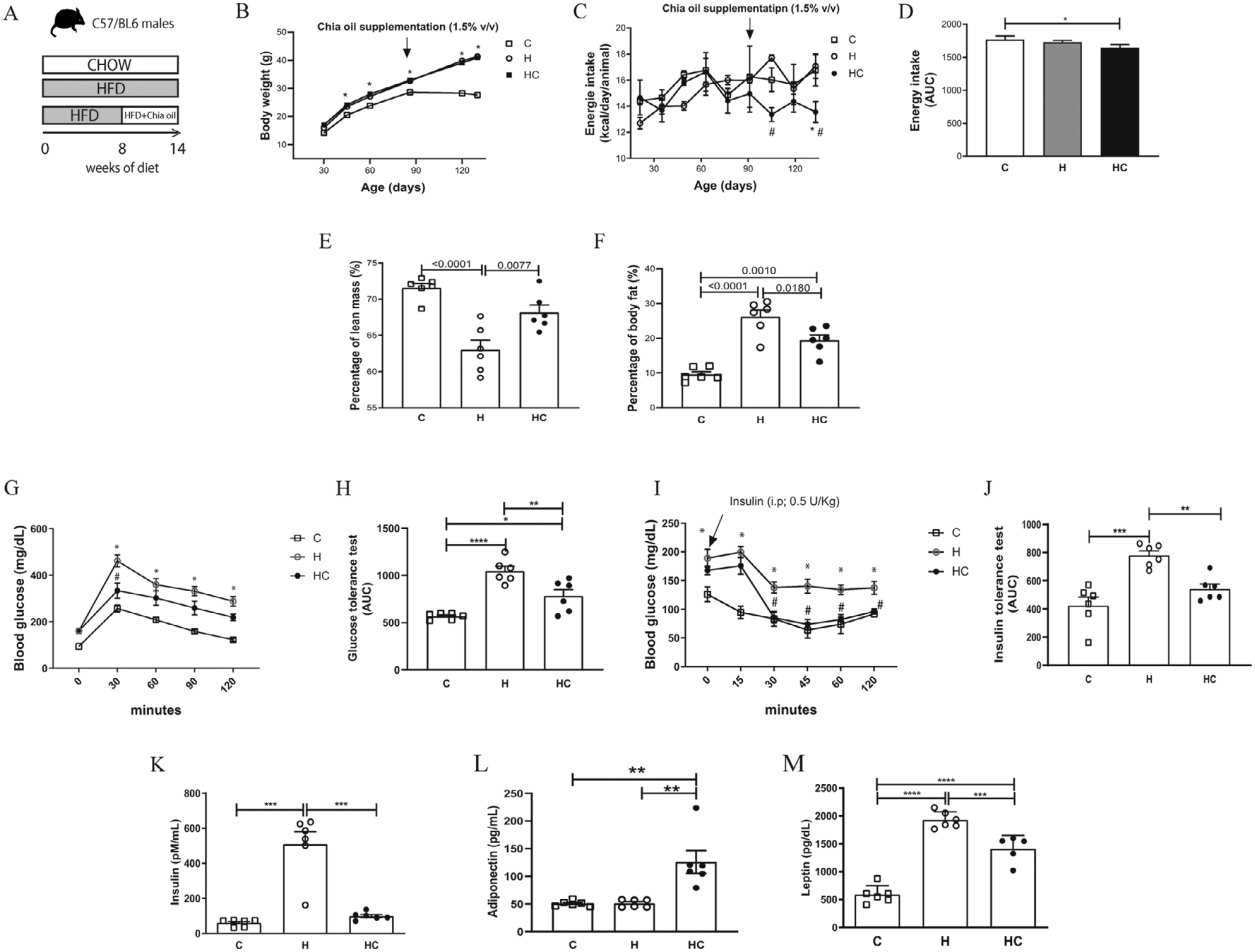
Dietary chia oil supplementation improves body composition and glucose homeostasis in obese mice. (A) Experimental design. (B) Body mass gain over time. (C) Energy intake curve. (D) Area under the curve (AUC) of energy intake. (E) Lean mass percentage. (F) Fat mass percentage as measured by NMR. (G) Intraperitoneal glucose tolerance test (ipGTT) and (H) its corresponding AUC. (I) Intraperitoneal insulin tolerance test (ipITT). (J) Corresponding AUC. (K) Fasting insulin levels. (L) Serum adiponectin. (M) Serum leptin concentrations. Data are presented as mean ± SEM. Statistical analyses were performed using two‐way RM ANOVA (B, C, G, and I) and one‐way ANOVA for endpoint measurements (D, E, F, H, J, K, L, and M), followed by appropriate post‐hoc tests as described in Experimental section. *p < 0.05, **p < 0.01, ***p <0,005, ****p < 0,001. *n* = 5–6 animals/group. C = Control; H = High‐fat diet; HC = High‐fat diet + chia oil.

Chia oil was administered as a dietary supplement incorporated into the high‐fat diet at a final concentration of 1.5% (w/w) from weeks 8 to 14 of the experimental protocol (Figure 1A). This dose was selected based on previous studies reporting metabolic benefits of chia oil in murine models (15,16), while allowing continuous dietary administration without gavage. Based on average food intake and body weight, chia oil supplementation corresponded to an estimated intake of ∼1.5 g chia oil/kg/day, equivalent to a human equivalent dose (HED) of ∼120 mg/kg/day, of which about 60% is α‐linolenic acid (∼70 mg/kg/day). This dose was selected not only for its ω‐3 content but also to achieve a balanced ω6/ω3 ratio in diet. The resulting dietary ratio was adjusted to approximately 4:1 (ω6/ω3), a ratio consistent with reported anti‐inflammatory benefits in human nutrition [17]. Additional details regarding diet formulation are provided in Tables S1–S3. Body weight and food intake were monitored throughout the intervention.

### 2.3 Body Composition Analysis

Body composition was assessed by TD‐NMR g (Minispec LF90, Bruker, Billerica, MA, USA). This method was selected over DEXA due to reproducibility, minimal animal stress, and absence of ionizing radiation. The device was calibrated using standard reference scans from a set of control mice (body weight: 25–35 g). Each animal was placed in a clear plastic cylinder (50 mm diameter) and immobilized without anesthesia using a tight‐fitting plunger. Scanning was performed for approximately 2 min per animal.

### 2.4 Glucose and Insulin Tolerance Test

For ipGTT, mice were fasted for 12 h and received 2 g/Kg glucose (Sigma‐Aldrich, St Louis, MO, USA). For ipITT, mice fasted for 4 h and injected with 0.5 U/Kg insulin (Humulin, Eli Lilly, Indianapolis, IN, USA). Blood glucose was measured via tail puncture using Accu‐Check active glucometer (Roche Diagnostics, Mannheim, Germany). Area under curves (AUC) were calculated as the total peak above the baseline at *t* = 0 min.

### 2.5 Plasma Hormones, Lipids, and Cytokines

Plasma insulin and adiponectin levels were measured using ELISA kits from MilliporeSigma (Burlington, MA, USA). Plasma leptin, TNF‐α, and IL‐6 were quantified using ELISA kits from Peprotech (Rocky Hill, NJ, USA) according to the manufacturer's instructions. Additionally, IL‐1β, IL‐10, and TNF‐α levels in PVAT supernatants were measured using ELISA kits from Peprotech. All assays were performed in duplicate following manufacturer's protocols.

### 2.6 Tissue Collection

Mice were euthanized under anesthesia (ketamine 50 mg/kg and xylazine 20 mg/kg). Liver and mesenteric PVAT were excised, weighed, snap‐frozen and stored at −80°C.

### 2.7 Hepatic Lipid Quantification

Fifty micrograms of liver were homogenized in isopropanol and centrifuged at 2000 g. Triglycerides were quantified using Bioclin K55 kit (Bioclin, Belo Horizonte, MG, Brazil) and normalized to protein levels using BCA protein assay kit (Thermo Fisher Scientific, Waltham, MA, USA). All measurements were performed in duplicate.

### 2.8 Histological Analysis

Liver and PVAT samples were fixed in 4% paraformaldehyde, paraffin‐embedded, and stained with hematoxylin–eosin (H&E). Images were captured at 20× magnification using an Olympus BX40 microscope. Adipocyte area and number were quantified using Adiposoft software.

### 2.9 Conditioned Medium Preparation From PVAT

PVAT fragments (50 mg) were incubated for 24 h in 1:1 DMEM/M199 medium. Supernatants were centrifuged and stored at −80°C.

### 2.10 Sample Preparation for Protein Analysis

Tissues were homogenized in RIPA buffer (Thermo Fisher Scientific, Waltham, MA, USA) with protease and phosphatase inhibitor cocktails (Roche Diagnostics, Manheim, Germany). Samples were centrifuged at 12,000 × *g* for 10 min at 4°C, and supernatants were used for protein quantification with BCA protein kit assay (Thermo Fisher Scientific, Waltham, MA, USA).

### 2.11 Western Blotting Analysis

Western blotting was performed as previously described [18]. Protein loading was standardized at 30–40 µg per lane. Primary and secondary antibodies are listed in Table S4. Blots represent biological replicates. Molecular weight markers were included in all figures.

### 2.12 RNA Extraction and Quantitative Real‐Time PCR

Total RNA from liver and PVAT were extracted using RNeasy Mini Kit (Qiagen, Hamburg, Germany). cDNA was synthesized using high‐capacity cDNA reverse transcription kit (Applied Biosystems, Foster City, CA, USA). Reactions used SYBR Green in a Rotor‐Gene Q system (Qiagen, Hilden, Germany). Melt‐curve analysis confirmed specificity and reaction efficiencies ranged from 95–105%. Gene expression was normalized to 18S and analyzed using the 2−ΔΔC_t_ method. Primers are listed in Table S5.

### 2.13 Blood Pressure and Heart Rate Measurements

Systolic, diastolic blood pressure, and heart rate were measured noninvasively using the CODA tail‐cuff system (Kent Scientific Corporation, Torrington, CT, USA). Mice were acclimated to the procedure for two weeks before measurements, which were performed weekly for 8 weeks.

### 2.14 Vascular Reactivity Studies

Mesenteric vascular beds (MVB) were isolated and perfused with physiological saline solutions. Vasoconstrictor responses to norepinephrine (0.1–1000 nM) and vasodilator responses to acetylcholine (1–1000 pmol) were assessed. Perfusion pressure was recorded using a PowerLab 4/30 system (ADInstruments, Dunedin, New Zealand).

### 2.15 Statistical Analysis

Statistical analyses were performed using GraphPad Prism 8.0 (GrahPad Software, San Diego, CA, USA). Data distribution were assessed using the Shapiro–Wilk normality test and a ROUT (*Q* = 1%) was applied to identify statistical outliers when appropriate. For comparisons among independent groups, normally distributed data were analyzed using one‐way ANOVA followed by Tukey's multiple‐comparison test. When normality assumptions were not met, the Kruskal–Wallis test followed by Dunn's post hoc test was applied. For longitudinal measurements, including body weight, food intake, GTT, ITT, systolic and diastolic blood pressure and heart rate, two‐way repeated‐measure (RM) ANOVA was applied, considering time and experimental group as fixed factors. Vascular reactivity curves were analyzed using two‐way ANOVA, considering agonist concentration and experimental group as independent factors. Effect sizes were calculated to quantify the magnitude of observed effects. For ANOVA‐based analyses, eta‐squared (η^2^) was reported, representing the proportion of total variance explained by each factor. For non‐parametric comparisons, epsilon‐squared (ε^2^) was calculated. Effect sizes were interpreted according to established benchmarks (small, moderate, or large) and reported in the corresponding Tables S6–S10. Data are presented as mean ± SEM. Statistical significance was set at *p* < 0.05.

## 3 Results

### 3.1 Chia oil Supplementation Attenuates Metabolic Dysregulation and Improves Body Composition in Obese Mice

The experimental design is illustrated in Figure 1A. Chia oil supplementation did not significantly alter body weight gain (Figure 1B) or caloric intake in obese mice (Figure 1C and D). However, it resulted in a marked increase in percent lean mass (*p* = 0.007) while percent fat mass decreased (*p* = 0.018) (Figure 1E and F, respectively). Additionally, chia oil improved glucose tolerance and insulin sensitivity (Figure 1G and I), reflected by reduced AUC values in ipGTT and ipITT. (Figure 1H and J). Fasting insulin decreased significantly (Figure 1K), while adiponectin increased (Figure 1L, *p* < 0.01) and leptin decreased (Figure 1M, *p* < 0.05).

Complementary statistical analyses provide the magnitude of the observed effects (Table S6), effect size analyses for longitudinal outcomes indicated that body weight gain was primarily driven by time (74% variance), while experimental group and time x group interaction contributed to the modulation of weight trajectory (11.9% and 4.1 %, respectively). In contrast, glucose and insulin tolerance tests showed substantial contributions of both time and experimental group. In GTT and ITT, the time factor reflects changes following intraperitoneal glucose or insulin administration, supporting a robust treatment‐dependent effect on glucose homeostasis. Food intake exhibited a greater contribution of the interaction term and subject‐related variability, reflecting heterogeneous individual responses over time.

### 3.2 Chia Oil Supplementation Improves Lipid Profile and Liver Damage in Obese Mice

**FIGURE 2:**
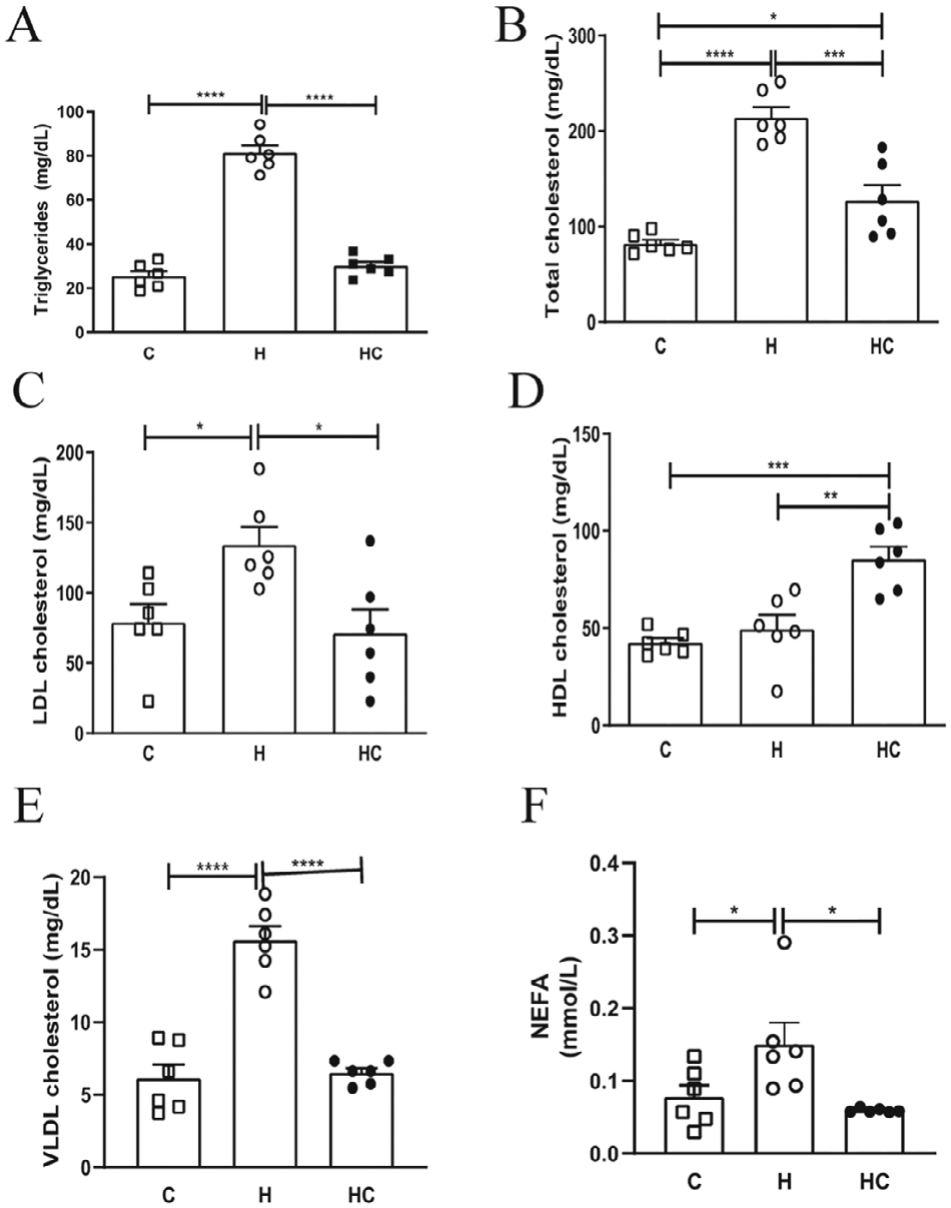
Chia oil supplementation improves circulating lipid profile in obese mice. (A) Serum triglyceride levels, (B) total cholesterol, (C) low‐density lipoprotein cholesterol (LDL‐c), (D) high‐density lipoprotein cholesterol (HDL‐c), (E) very‐low‐density lipoprotein cholesterol (VLDL‐c), and (F) nonesterified fatty acids (NEFA). Data are presented as mean ± SEM. Statistical analyses were performed using one‐way ANOVA followed by appropriate post hoc tests as described in experimental section. *p < 0.05, **p < 0.01, ***p <0,005, ****p<0,001. *n* = 5–6 animals/group. C = Control; H = High‐fat diet; HC = High‐fat diet + chia oil.

**FIGURE 3:**
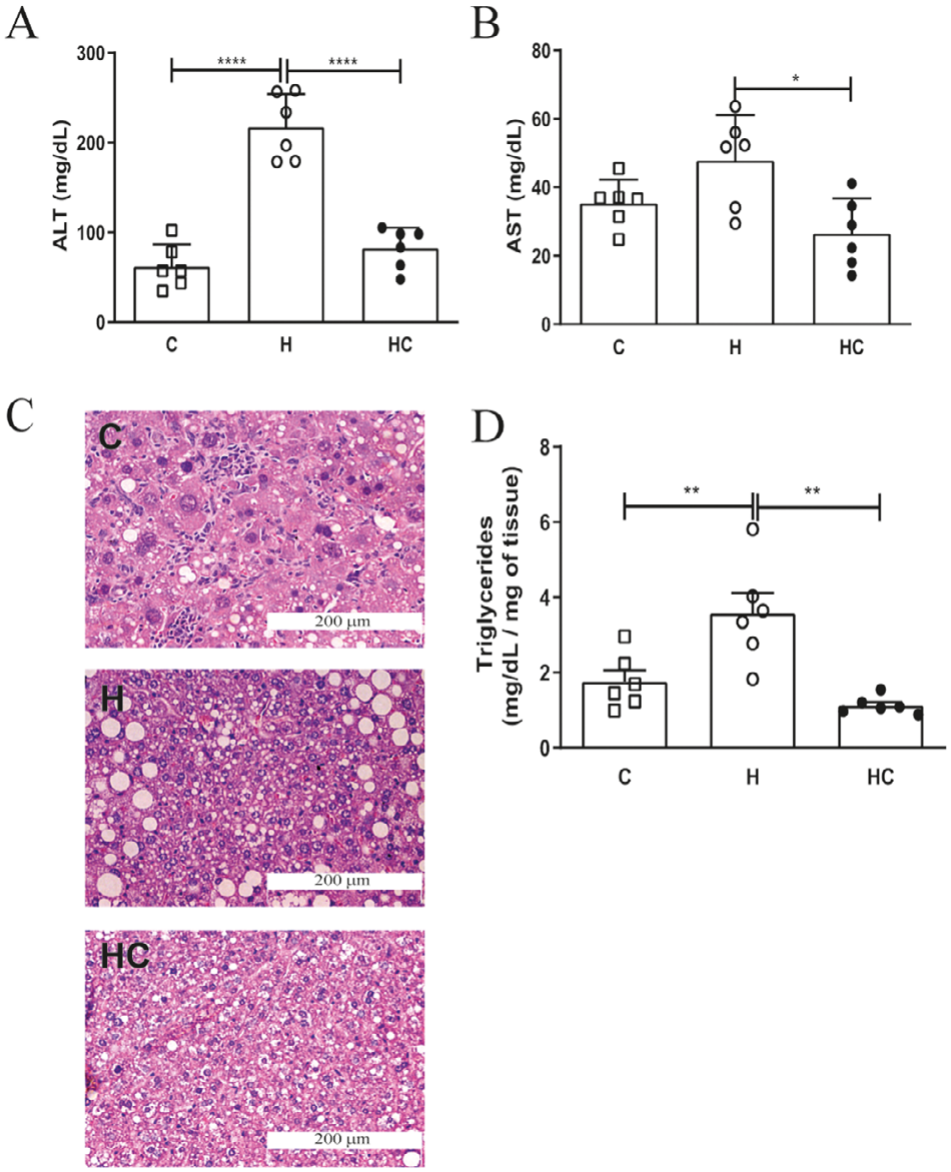
Chia oil supplementation reduces hepatic steatosis and lipid accumulation in obese mice. (A) Serum levels of alanine aminotransferase (ALT) and (B) aspartate aminotransferase (AST). (C) Representative hematoxylin and eosin (H&E)‐stained liver sections from each group, showing hepatic architecture and steatosis (magnification: 20×). (D) Quantification of hepatic triglyceride content. Data are presented as mean ± SEM. Statistical analyses were performed using one‐way ANOVA followed by appropriate post hoc tests as described in experimental section. *p < 0.05, **p < 0.01, ***p <0,005, ****p<0,001. *n* = 6 animals/group. C = Control; H = High‐fat diet; HC = High‐fat diet + chia oil.

Chia oil significantly reduced plasma triglycerides, total cholesterol, LDL, VLDL, and NEFAs (Figure 2A–E, *p* < 0.05 for all), while increasing HDL levels (Figure 2F, *p* < 0.05). The improvement in lipid profiles was paralleled by a reduction (*p* <0.05) in plasma ALT and AST levels (Figure 3A and B, respectively), indicating decreased hepatic injury. Histopathological analysis confirmed reduced macrovesicular steatosis, hepatocyte ballooning, decreased inflammatory infiltration (Figure 3C) and hepatic triglyceride accumulation in chia oil‐treated mice (Figure 3D), suggesting a protective role of chia oil against obesity‐induced liver damage. No signs of significant fibrosis were observed in the groups.

Endpoint analyses revealed consistently large effect sizes across body composition, circulating metabolic parameters—including adipokines and lipid profile—and hepatic outcomes such as ALT, AST and hepatic triglyceride content (Table S7), indicating that a substantial proportion of variance in these endpoints was explained by the experimental condition.

### 3.3 Chia Oil Modulates Hepatic Lipid Metabolism and Inflammatory Pathways

**FIGURE 4:**
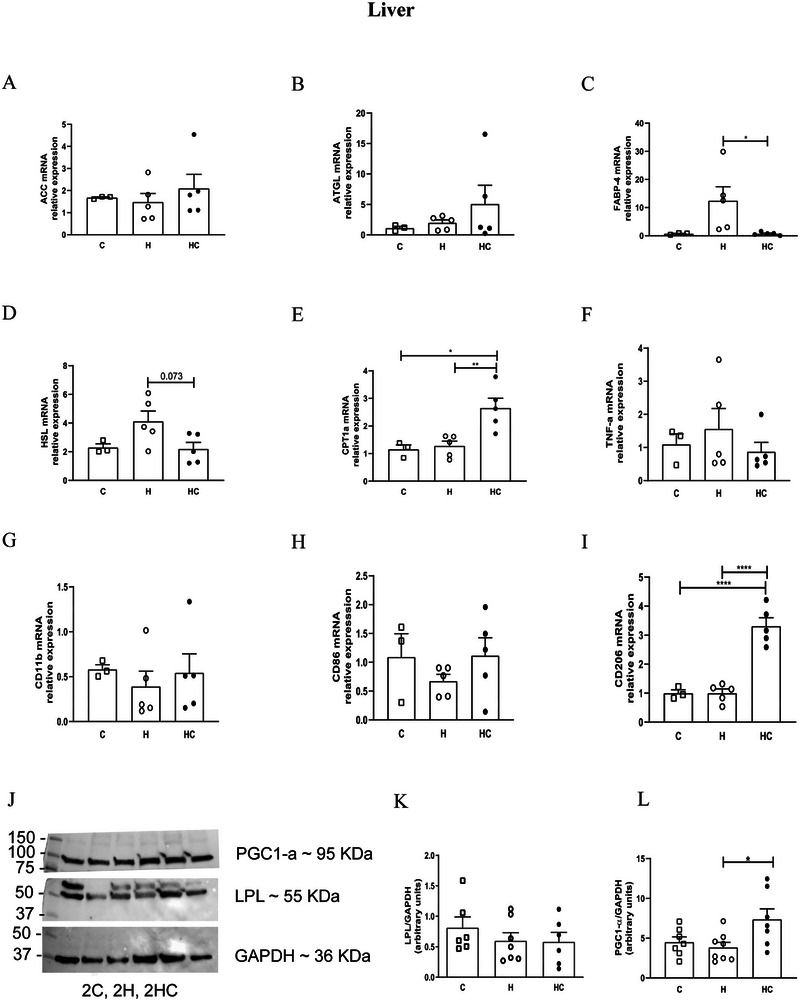
Chia oil supplementation modulates hepatic expression of genes and proteins involved in lipid metabolism and inflammation in obese mice. Relative mRNA expression of (A) acetyl‐CoA carboxylase (ACC), (B) adipose triglyceride lipase (ATGL), (C) fatty acid‐binding protein 4 (FABP4), (D) hormone‐sensitive lipase (HSL), and (E) carnitine palmitoyltransferase 1 alpha (CPT‐1α). Hepatic expression of inflammatory and macrophage polarization markers: (F) TNF‐α, (G) CD11b, (H) CD86, and (I) CD206. (J‐L) Protein expression of lipoprotein lipase (LPL) and PGC‐1α assessed by Western blotting. Gene expression levels were normalized to 18S rRNA and are expressed relative to the control group. Anti‐GAPDH antibody was used as the loading control in western blotting. Data are presented as mean ± SEM. Statistical analyses were performed using one‐way ANOVA followed by appropriate post‐hoc tests as described in experimental section. *p < 0.05, **p < 0.01. *n* = 3–7 animals/group. C = Control; H = High‐fat diet; HC = High‐fat diet + chia oil.

No significant changes were observed in the gene expression of ACC and ATGL as well as in LPL protein expression between the experimental groups (Figure 4A, B, J, and K, respectively). Interestingly, chia oil supplementation significantly decreased FABP‐4 gene expression (*p* <0.05) in the liver of obese mice (Figure 4C), while. HSL gene expression exhibited a trend toward reduction (Figure 4D, *p* = 0.073). Chia oil supplementation increased CPT‐1α expression (Figure 4E), indicative of enhanced mitochondrial fatty acid oxidation. Additionally, increased protein expression of PGC‐1α suggested improved mitochondrial biogenesis (Figure 4 J and L). Notably, while TNF‐α, CD11b, and CD86 expression remained unchanged (Figure 4F–H), chia oil supplementation upregulated CD206 (Figure 4I), indicating early signals of anti‐inflammatory polarization.

Effect size analysis revealed large effects for FABP4, CPT‐1α, CD206, and PGC1‐α expression (*η* ^2^ = 0.31–0.88), while lipogenic and inflammatory markers such as ACC, ATGL, TNF‐α, and CD11b showed small effects (*η* ^2^< 0.20), indicating selective modulation of hepatic metabolic and macrophage‐related pathways by chia oil (Table S8).

### 3.4 Chia Oil Modulates Mesenteric PVAT Remodeling and Inflammatory Profile

**FIGURE 5:**
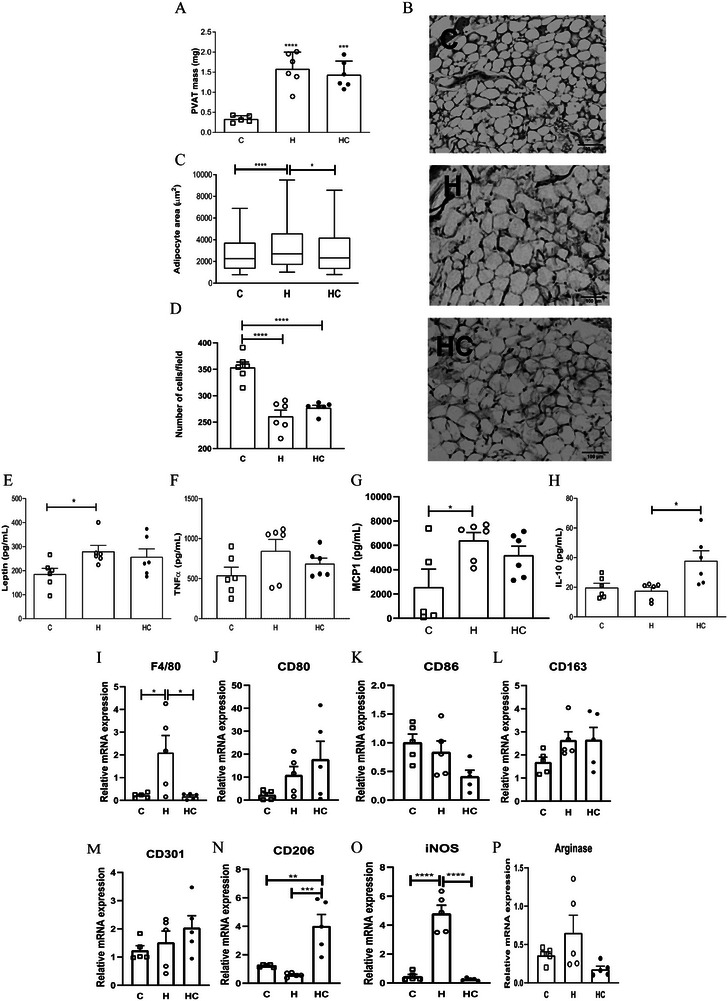
Chia oil supplementation promotes anti‐inflammatory remodeling of mesenteric perivascular adipose tissue (PVAT) in obese mice. (A) Mass of mesenteric PVAT. (B) Representative hematoxylin and eosin (H&E)‐stained sections of PVAT (magnification: 20×). (C) Quantification of adipocyte area. (D) Number of adipocytes. (E–H) Secretion levels of leptin, TNF‐α, MCP‐1 and IL‐1 measured by ELISA. (I) Gene expression of macrophage marker F4/80. (J‐M) mRNA expression of polarization markers CD80, CD86, CD163, and CD301. (N) mRNA expression of CD206, a marker of M2 macrophages. (O), (P) mRNA expression of inducible nitric oxide synthase (iNOS) and arginase‐1. Gene expression levels were normalized to 18S rRNA and expressed relative to the control group. Data are presented as mean ± S.E.M. Statistical analyses were performed using one‐way ANOVA followed by appropriate post‐hoc tests as described in experimental section. *p < 0.05, **p < 0.01, ***p<0.005, ****p<0.0001. *n* = 5–6 animals/group. C = Control; H = High‐fat diet; HC = High‐fat diet + chia oil.

Chia oil supplementation did not significantly alter the mass of mesenteric PVAT in obese mice (Figure 5A), but significantly decreased volume adipocyte (Figure 5B and C, *p* < 0.05), suggesting an effect on adipocyte morphology. Obesity reduced adipocyte number per field (*p* <0.0001), consistent with hypertrophy, while chia oil supplementation did not alter this parameter (Figure 5D). Moreover, chia oil supplementation did not affect leptin, TNF‐α or MCP1 secretion in mesenteric PVAT (Figure 5E–G), but markedly increased IL‐10 secretion (Figure 5H, *p* < 0.05). This effect was further supported by a significant reduction in F4/80 expression (Figure 5I, *p* < 0.05). Additionally, while no significant differences were observed in the expression of CD80, CD86, CD163, and CD301 (Figure 5J–M), a notable increase in CD206 gene expression was detected in mesenteric PVAT of obese mice supplemented with chia oil (Figure 5N, *p* < 0.005). This elevation in CD206, a marker for M2 macrophages, was accompanied by a significant downregulation of iNOS (p < 0.001), but not of arginase expression (Figure 5O and P, respectively), reinforcing the potential role of chia oil in modulating adipose tissue inflammation and dysfunction. Effect size analysis revealed that most outcomes in PVAT exhibited large global effects, while adipocyte area displayed only a small effect size (*η* ^2^ = 0.029) (Table S9).

### 3.5 Chia Oil Supplementation Enhances Endothelial Function in Obesity

**FIGURE 6:**
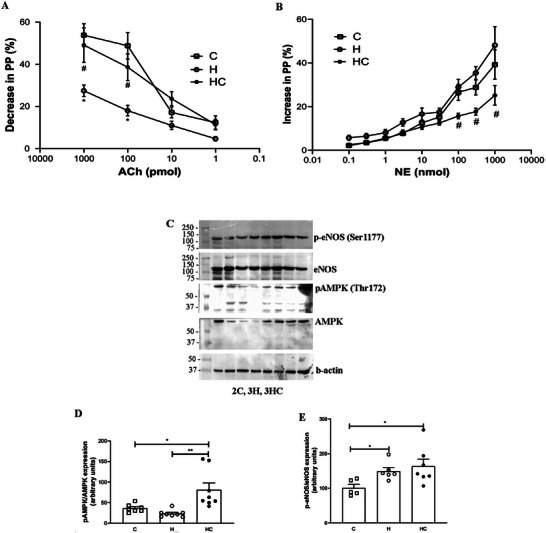
Chia oil supplementation improves vascular function and enhances AMPK signaling in mesenteric PVAT of obese mice. (A) Endothelium‐dependent vasodilation in response to acetylcholine (Ach) and (B) vasoconstriction response to norepinephrine (NE) in isolated mesenteric vascular beds. (C‐E) Protein expression of total and phosphorylated AMPK (Thr172) and total and phosphorylated eNOS (Ser1177) in mesenteric perivascular adipose tissue (PVAT), analyzed by Western blotting. Anti‐β‐actin antibody was used as the loading control. Data are presented as mean ± S.E.M. Statistical analyses were performed using two‐way RM ANOVA (A and B) and one‐way ANOVA for endpoint measurements (D and E), followed by appropriate post‐hoc tests as described in experimental section. *p < 0.05, **p < 0.01; #*p* < 0.05 vs. H. *n* = 5–6 animals/group. C = Control; H = High‐fat diet; HC = High‐fat diet + chia oil.

Obese mice exhibited impaired endothelium‐dependent vasodilation in response to acetylcholine (ACh), consistent with endothelial dysfunction (Figure 6A, *p* < 0.05). Chia oil supplementation restored vasodilatory capacity (Figure 6A, *p* < 0.05) and attenuated norepinephrine (NE)‐induced vasoconstriction (Figure 6B, *p* < 0.05), indicating a vasoprotective effect. Effect size analysis showed that vasorelaxation was predominantly driven by the agonist factor, which accounted for a large proportion of the variance (*η* ^2^ = 0.47), whereas the group effect was moderate (*η* ^2^ = 0.12) and the interaction effect was small (*η* ^2^ = 0.04) (Table S10). Similarly, vasoconstriction responses were largely explained by the agonist factor (*η* ^2^ = 0.60), with comparatively modest contributions from group (*η* ^2^ = 0.06) and interaction effects (*η* ^2^ = 0.04).

These functional improvements were accompanied by increased AMPK phosphorylation at Thr172 in PVAT, a key regulator of endothelial homeostasis (Figure 6C and D, *p* < 0.01). Although obesity increased eNOS phosphorylation at Ser1177, chia oil supplementation did not significantly modify this phosphorylation profile (Figure 6C and E, *p* < 0.05). Consistent with these findings, p‐AMPK/AMPK displayed a large effect size (*η* ^2^ = 0.58), indicating that a substantial proportion of the variance in AMPK activation was explained by the experimental condition (Table S9). In contrast, p‐eNOS/eNOS exhibited a statistically significant but more modest group effect, as determined by non‐parametric analysis, suggesting limited modulation of nitric oxide signaling.

Chia oil supplementation did not induce significant changes in systemic blood pressure or heart rate (Figure S1). Effect size analysis revealed a moderate time‐dependent effect on systolic blood pressure (*η* ^2^ = 0.15), while group and interaction effects were moderate (*η* ^2^ = 0.06). Diastolic blood pressure showed uniformly small effect sizes across all factors (*η* ^2^ ≤ 0.11), indicating minimal impact of the dietary intervention. In contrast, heart rate exhibited a moderate group effect (*η* ^2^ = 0.13) and a large time × group interaction effect (*η* ^2^ = 0.18), suggesting dynamic modulation of cardiac autonomic regulation over time (Table S10).

## 4 Discussion

Our findings provide further evidence supporting the beneficial effects of chia oil supplementation in obesity‐associated metabolic dysfunction by modulating hepatic lipid metabolism, attenuating PVAT inflammation and restoring vascular responsiveness. Our results expand previous evidence that omega‐3 fatty acids, particularly ALA from chia oil, act not only systemic lipid metabolism but also on tissue‐specific mechanisms relevant to early MASLD progression and vascular homeostasis [15, 16].

Chia oil supplementation improved lipid metabolism by reducing plasma triglycerides, cholesterol, NEFA and hepatic fat accumulation, accompanied by decreased FABP4 expression and enhanced CPT‐1α and PGC‐1α levels. These results suggest improved mitochondrial fatty‐acid oxidation, consistent with prior evidence that omega‐3 fatty acids enhance hepatic oxidative capacity [19, 20, 21, 22]. Importantly, effect size analysis revealed large effects for CPT‐1α, PGC‐1α, and FABP4 expression, indicating that a substantial proportion of the variance in these hepatic metabolic markers was explained by the experimental condition.

We also explored the effects of chia oil on inflammatory processes in the liver of obese mice. Notably, although key inflammatory markers (TNF‐α, CD11b, CD86, and CD206) in the liver did not differ significantly between obese and control animals, this is consistent with relatively mild phenotype of early‐stage steatosis induced by high‐fat diet. The model did not reach the threshold of significant hepatic inflammation and we explicitly acknowledge this limitation. Consistently, effect size estimates for classical inflammatory markers were small, supporting the absence of overt hepatic inflammatory activation. Still, the observed metabolic improvements remain relevant to MASLD prevention at earlier disease stages.

Interestingly, chia oil supplementation increased the gene expression of CD206 in the liver of obese mice. CD206 is a classical marker of alternatively activated (M2‐like) macrophages, which are associated with tissue repair and resolution of inflammation [23, 24]. Although our obesity model did not induce marked hepatic inflammation, a limitation characteristic of early‐stage steatosis, the very large effect size observed for CD206 expression suggests that chia oil supplementation may still promote a shift toward a more anti‐inflammatory or reparative macrophage phenotype within the hepatic environment. This subtle immunological modulation is consistent with the observed reduction in hepatic steatosis and enhancement of mitochondrial oxidative pathways and may represent an early protective response prior to the establishment of overt inflammatory injury.

In mesenteric PVAT, chia oil supplementation significantly decreased adipocyte hypertrophy and reduced F4/80 expression, indicating both improved adipocyte morphology and lower macrophage infiltration. These structural changes were accompanied by increased IL‐10 secretion and higher CD206 expression, together with a reduction in iNOS levels and no significant alteration in arginase‐1 expression. Importantly, effect size analysis demonstrated that the experimental condition explained a substantial proportion of the variance across inflammatory, secretory and macrophage‐related markers in PVAT, even in cases where statistical significance was not reached. Taken together, these findings indicate that chia oil modulates the PVAT remodeling toward a regulatory and anti‐inflammatory profile, rather than inducing a fully polarized M2 phenotype.

Functionally, chia oil supplementation exerted vasoprotective effects in obese mice, restoring endothelium‐dependent vasodilation and attenuating vasoconstriction responses without significantly altering systemic blood pressure. Effect size analysis reinforced these findings by showing that vascular reactivity was primarily driven by agonist‐dependent mechanisms, while group‐related effects were moderate and interaction effects remained small. The functional improvement was accompanied by robust activation of AMPK in PVAT, as indicated by a large effect size for p‐AMPK/AMPK, supporting a central role for AMPK signaling in mediating the vascular benefits of chia oil.

In contrast, changes in eNOS phosphorylation were modest, suggesting that AMPK‐related pathways may precede or outweigh direct modulation of nitric oxide signaling at this disease stage. Notably, cardiovascular parameters displayed limited sensitivity to dietary intervention, with only heart rate showing a dynamic time × group interaction, indicating subtle autonomic modulation rather than over hemodynamic changes. Collectively, these data suggest that chia oil improves vascular function primarily through local metabolic and signaling adaptations, which may represent early protective mechanisms preceding systemic cardiovascular remodeling.

Although the relatively modest sample size represents an inherent limitation of mechanistic in vivo studies, the large and consistent effect sizes observed across metabolic, inflammatory, and vascular endpoints reinforce the biological relevance of the present findings. Notably, treatment effects were evident across independent yet interconnected physiological systems, including hepatic metabolism, adipose tissue remodeling, vascular function, and cardiometabolic regulation. Collectively, these results indicate that chia oil supplementation induces coordinated protective adaptations that may mitigate early obesity‐associated dysfunction and delay the progression toward more advanced metabolic and cardiovascular disease.

## 5 Conclusion

Dietary chia oil supplementation attenuates key features of early obesity‐associated metabolic dysfunction, including hepatic steatosis, PVAT inflammation and endothelial impairment. By enhancing lipid oxidation, reducing inflammatory activation, and improving vascular responsiveness, ALA‐rich chia oil emerges as a promising functional food component for MASLD prevention and metabolic improvement.

## Funding

This work was supported by the Conselho Nacional de Desenvolvimento Científico e Tecnológico (Project N°: 444675/2023‐0; 402918/2023‐1), Fundação Carlos Chagas Filho de Amparo à Pesquisa do Estado do Rio de Janeiro (Project N°: SEI‐260003/001184/2023; SEI‐260003/005820/2024). The funders had no role in the study design, data collection, data analysis, preparation of the manuscript, or the decision to publish.

## Conflicts of Interest

The authors declare no conflicts of interest.

## Supporting information




**Supporting File**: mnfr70469‐sup‐0001‐SuppMat.docx.

## Data Availability

The data that support the findings of this study are available from the corresponding author upon reasonable request.
