# Supplementary material for: Chia Oil Supplementation Attenuates Obesity‐Induced Hepatic Steatosis, PVAT Inflammation, and Endothelial Dysfunction in Mice
Source: Mol Nutr Food Res. 2026 Apr 17;70:e70469. doi: 10.1002/mnfr.70469 (PMC13088004; doi:10.1002/mnfr.70469)
Supplement: Supplementary file 1 — Supporting File: mnfr70469‐sup‐0001‐SuppMat.docx. [file MNFR-70-e70469-s001.docx]

**Chia oil supplementation attenuates obesity-induced hepatic steatosis, PVAT inflammation and endothelial dysfunction in mice**

Agatha de Assis-Ferreira^1^*, Gabrielly Muniz-Cassuce^1^*, Thaís Fonte-Faria^18^, Thamiris de Souza^2^, Marta Citelli^2^, Lenize Costa Reis Marins de Carvalho^3^, Dayane Teixeira Ognibene^3^, Graziele Freitas de Bem^3^, Angela Castro Resende^3^, Christina Barja-Fidalgo^1^, Simone Vargas da Silva^1#^.

1 Departamento de Biologia Celular, Universidade do Estado do Rio de Janeiro, RJ, Brazil.

2 Instituto de Nutrição, Departamento de Nutrição Básica e Experimental, Universidade do Estado do Rio de Janeiro, RJ, Brazil.

3 Departamento de Farmacologia e Psicobiologia, Universidade do Estado do Rio de Janeiro, RJ, Brazil.

# Correspondence: [simonevargasdasilva@gmail.com](mailto:simonevargasdasilva@gmail.com)

**Table of contents:**

Table S1: Diet composition

Table S2: Macronutrient composition and energy density of experimental diets

Table S3: Fatty acid composition and ω-6:ω-3 ratio of experimental diets

Table S4: Antibodies used in the study

Table S5: Genes analyzed by qPCR

Table S6: Effect size estimates for longitudinal body weight, food intake and glucose homeostasis

Table S7: Effect size estimates for whole-body composition, plasma metabolic profile and hepatic lipid accumulation

Table S8: Effect size analysis of hepatic gene and protein expression

Table S9: Global effect size estimates for PVAT outcomes

Table S10: Effect size analysis of vascular reactivity and cardiovascular parameters

Supplemental figure S1: Chia oil supplementation does not alter systemic blood pressure or heart rate in obese mice.

**Table S1. Diet composition**

| Ingredient | C | H | HC |
| --- | --- | --- | --- |
| Corn starch | 39.0 | 18.5 | 18.5 |
| Dextrose | 13.2 | 6.1 | 6.1 |
| Casein | 20.0 | 20.0 | 20.0 |
| Sucrose | 10.0 | 10.0 | 10.0 |
| Soybean oil | 7.0 | 7.0 | 7.0 |
| Cellulose | 5.0 | 5.0 | 5.0 |
| Mineral mix | 3.5 | 3.5 | 3.5 |
| Vitamin mix | 1.0 | 1.0 | 1.0 |
| Cystine | 0.3 | 0.3 | 0.3 |
| Choline | 0.25 | 0.25 | 0.25 |
| Lard | 0.0 | 13.3 | 13.3 |
| Chia oil | 0.0 | 0.0 | 1.5 |

Legend: Ingredient composition of the control diet (C), high-fat diet (H) and high-fat diet supplemented with chia oil (HC). Values are expressed as percentage of total diet weight (g per 100 g of diet). The H diet was enriched with lard (13.3%) to induce obesity, while the HC diet maintained the same high-fat composition with the addition of 1.5% (w/w) chia oil. All other macronutrient and micronutrient components were kept identical among groups to ensure that differences between H and HC diets were attributable specifically to chia oil supplementation.

**Table S2. Macronutrient composition and energy density of experimental diets**

| Parameter | C | H | HC |
| --- | --- | --- | --- |
| Carbohydrates (g/100 g) | 63.0 | 34.0 | 34.0 |
| Proteins (g/100 g) | 20.0 | 20.0 | 20.0 |
| Lipids (g/100 g) | 7.0 | 20.2 | 21.7 |
| Energy (kcal/g) | 3.96 | 4.73 | 4.81 |

Legend: Macronutrient distribution and calculated energy density of the control (C), high-fat (H), and high-fat supplemented with chia oil (HC) diets. Values are expressed as grams per 100 g of diet (g/100 g). Energy density (kcal/g) was calculated based on standard factors (4 kcal/g for carbohydrates and proteins; 9 kcal/g for lipids).

**Table S3. Fatty acid composition and ω-6:ω-3 ratio of experimental diets**

| Fatty acid | C | H | HC |
| --- | --- | --- | --- |
| Palmitic (16:0) | 0.75 | 3.46 | 3.55 |
| Stearic (18:0) | 0.23 | 1.67 | 1.72 |
| Oleic (18:1 ω-9) | 1.60 | 6.76 | 6.85 |
| Linoleic (18:2 ω-6) | 3.76 | 6.34 | 8.4 |
| α-linolenic (18:3 ω-3) | 0.40 | 0.55 | 2.05 |
| SFA | 1.06 | 6.31 | 6.45 |
| PUFA | 4.20 | 12.14 | 13.36 |
| ω-6:ω-3 ratio | 9.40 | 11.52 | 4.1 |

Legend: Fatty acid profile of the control (C), high-fat (H) and high-fat supplemented with chia oil (HC) diets expressed as grams per 100 g of diet (g/100 g). SFA – saturated fatty acids, PUFA – polyunsaturated fatty acids. Incorporation of 1.5% chia oil in the HC diet increased α- linolenic acid content and reduced the ω-6:ω-3 ratio compared to the H diet, resulting in a more balanced fatty acid profile consistent with nutritional recommendations favoring lower ω-6:ω-3 ratios.

**Table S4: Antibodies used in the study**

| **Name** | **Host** | **Company/Catalog** | **Application and Dilution** |
| --- | --- | --- | --- |
| **AMPK α 1/2** | Mouse | Santa Cruz (sc-74461) | WB; 1:500 |
| **p-AMPK (Thr 172)** | Rabbit | Santa Cruz (sc-33524) | WB; 1:500 |
| **Beta-actin** | Rabbit | Cell signaling (4970) | WB; 1:1000 |
| **eNOS** | Rabbit | Invitrogen (PA1-037) | WB; 1:1000 |
| **p-eNOS (Ser1177)** | Rabbit | Invitrogen (MA5-14957) | WB; 1:500 |
| **GAPDH** | Mouse | Invitrogen (MA1-16757 | WB; 1:1000 |
| **LPL** | Rabbit | Sigma-Aldrich (SAB5700273) | WB; 1:1000 |
| **PGC-1α** | Rabbit | Invitrogen (PA5-72948) | WB; 1:500 |
| **Anti-rabbit IgG, HRP-linked Antibody** | Goat | Cell signaling (7074) | WB; 1:5000 |
| **Anti-mouse IgG, HRP-linked Antibody** | Horse | Cell signaling (7076) | WB; 1:5000 |

Abbreviations: WB, western blot

**Table S5: Genes analyzed by qPCR**

| **Gene Symbol** | **Function / Description** | **Access number of Gene bank** | **Catalog number/Sequence 5’-3’** |
| --- | --- | --- | --- |
| **rn18S** | Housekeeping gene for normalization | NR003278 | QT02448075 |
| **ACC** | Acetyl-CoA Carboxylase – lipogenesis | NM133360 | QT01554441 |
| **Arginase-1** | Urea cycle enzyme – M2 macrophage marker | NM007482 | F: CAGTGTGGTGCTGGGTGGAGACCA  R: AGGGTGGACCCTGGCGTGGC |
| **ATGL** | Adipose Triglyceride Lipase – lipolysis | NM025802 | QT00111846 |
| **CD11b** | Macrophage integrin – pan-macrophage marker | NM008401.2 | F: CTGGCTTTAGACCCTGTCCG  R: GCCTGCGTGTGTTGTTCTTT |
| **CD163** | Scavenger receptor – M2 macrophage marker | NM001170395.1 | F: ATCCTCGGGGGTCATTCAGAGG  R: GCTGGCTGTCCTGTCAAGGCT |
| **CD206** | M2 macrophage marker – anti-inflammatory | NM008625.2 | F: ATGGATTGCCCTGAACAGCA  R: TGTACCGCACCCTCCATCTA |
| **CD301** | M2 macrophage marker – immune regulation | NM001204252 | F: GGAATCCTCCTACCCGGTCT  R: ACCCAGCTCAAACACAATCCT |
| **CD80** | Co-stimulatory molecule – M1 macrophage marker | NM009855 | F: ACAACAGCCTTACCTTCGGG  R: TTTGCAGAGCCAGGGTAGTG |
| **CD86** | Co-stimulatory molecule – M1 macrophage marker | NM019388 | F: CAGACGCGTAAGAGTGGCTC  R: TGGGTGCTTCCGTAAGTTCTG |
| **CPT-1α** | Carnitine Palmitoyltransferase 1α – fatty acid oxidation | NM013495 | F: CGCTCATTCCGCCGCCGCCGT  R: GGCCACAGCTTGGTGAGCCTCTGCC |
| **F4/80** | Pan-macrophage marker – infiltration | X93328.1 | F: CGGCAGCACATGCAATCTCA  R: ACATTACCATCCCCCTTCCAC |
| **FABP4** | Fatty Acid Binding Protein 4 – fatty acid transport | NM024406 | QT00091532 |
| **HSL** | Hormone Sensitive Lipase – lipolysis | NM001039507 | QT00169057 |
| **iNOS** | Nitric oxide synthase2, inducible – M1 macrophage marker | NM010927 | QT01535800 |
| **TNF-α** | Tumor Necrosis Factor alpha – inflammation marker | NM013693 | QT00104006 |

Abbreviations: F – forward; R – reverse

Legend: Primers used for gene expression analysis by quantitative real-time PCR (qPCR). Primers labeled with “QT” codes were purchased as QuantiTect Primer Assays from Qiagen® (Hilden, Germany), optimized for SYBR Green-based detection. Primers with specified forward (F) and reverse (R) sequences were custom-designed and synthesized by Exxtend® – Molecular Biology (São Paulo, SP, Brazil) based on mRNA sequences available in GenBank. The rn18S gene was used as the endogenous control for normalization.

**Table S6: Effect size estimates for longitudinal body weight, food intake and glucose homeostasis**

| **Figure / Panel** | **Outcome** | **Statistical model** | **Time (% variance)** | **Group (% variance)** | **Time × Group (% variance)** | **Subject (% variance)** | **Interpretation** |
| --- | --- | --- | --- | --- | --- | --- | --- |
| Fig. 1B | **Body weight** | Two-way RM ANOVA | 74.4 | 11.9 | 4.1 | Not applicable | Time-dominant weight gain with significant group modulation |
| Fig. 1C | **Food intake (Kcal/day/animal)** | Two-way RM ANOVA | 17 | 6.9 | 23.2 | 15.4 | Moderate time-dependent modulation with high inter individual variability |
| Fig. 1G | **Glucose tolerance test (GTT)** | Two-way RM ANOVA | 44.9 | 34.4 | 3.6 | 12.1 | Time- and group-driven glycemic response |
| Fig. 1I | **Insulin tolerance test (ITT)** | Two-way RM ANOVA | 35.9 | 35 | 7.3 | 14.4 | Strong group and time effects on insulin sensitivity |

Legend: Effect size was estimated as the percentage of total variance explained by each source of variation in two-way repeated measures ANOVA models. Time, experimental group and their interaction were evaluated as fixed effects. Subject-related variance is reported when explicitly quantified by the model; for body weight, subject variance is incorporated into the residual term and is therefore indicated as not applicable. Effect size magnitude was interpreted according to conventional thresholds for eta-squared (η²): small (<0.06), moderate (0.06–0.13), and large (≥0.14).

**Table S7: Effect size estimates for whole-body composition, plasma metabolic profile and hepatic lipid accumulation**

| **Figure / Panel** | **Outcome** | **Sample** | **Statistical model** | **Effect size metric** | **Effect size value** | **Interpretation** |
| --- | --- | --- | --- | --- | --- | --- |
| Fig. 1E | Body lean mass (%) | Whole body | One-way ANOVA | η² | 0.70 | Large |
| Fig. 1F | Body fat mass (%) | Whole body | One-way ANOVA | η² | 0.80 | Large |
| Fig. 1K | Insulin | Blood | One-way ANOVA | η² | 0.82 | Large |
| Fig. 1L | Adiponectin | Blood | One-way ANOVA | η² | 0.64 | Large |
| Fig. 1M | Leptin | Blood | One-way ANOVA | η² | 0.92 | Large |
| Fig. 2A | Triglycerides | Blood | One-way ANOVA | η² | 0.95 | Large |
| Fig.2B | Total cholesterol | Blood | One-way ANOVA | η² | 0.82 | Large |
| Fig. 2C | LDL cholesterol | Blood | One-way ANOVA | η² | 0.43 | Large |
| Fig. 2D | HDL cholesterol | Blood | One-way ANOVA | η² | 0.67 | Large |
| Fig. 2E | VLDL cholesterol | Blood | One-way ANOVA | η² | 0.86 | Large |
| Fig. 2F | NEFA | Blood | One-way ANOVA | η² | 0.44 | Large |
| Fig. 3A | ALT | Blood | One-way ANOVA | η² | 0.87 | Large |
| Fig. 3B | AST | Blood | One-way ANOVA | η² | 0.46 | Large |
| Fig. 3C | Triglycerides | Liver | One-way ANOVA | η² | 0.62 | Large |

Legend: Effect size was calculated as eta-squared (η²) for endpoint outcomes analyzed by one-way ANOVA and is expressed as the proportion of total variance explained by the experimental group. All variables were measured at the end of the intervention period. Interpretation of effect size magnitude follows conventional thresholds for ANOVA-based measures, with all outcomes exhibiting large effects. Interpretation of effect size magnitude followed established benchmarks for eta-squared (η²): small (<0.06), moderate (0.06–0.13), and large (≥0.14).

**Table S8: Effect size analysis of hepatic gene and protein expression**

| **Figure /Panel** | **Outcome** | **Sample** | **Statistical model** | **Effect size metric** | **Effect size value** | **Interpretation** |
| --- | --- | --- | --- | --- | --- | --- |
| Fig. 4A | ACC mRNA | Liver | One-way ANOVA | η² | 0.08 | Moderate |
| Fig. 4B | ATGL mRNA | Liver | One-way ANOVA | η² | 0.16 | Large |
| Fig. 4C | FABP4 mRNA | Liver | One-way ANOVA | η² | 0.55 | Large |
| Fig. 4D | HSL mRNA | Liver | One-way ANOVA | η² | 0.43 | Large |
| Fig. 4E | CPT1α mRNA | Liver | One-way ANOVA | η² | 0.66 | Large |
| Fig. 4F | TNF-α mRNA | Liver | One-way ANOVA | η² | 0.11 | Moderate |
| Fig. 4G | CD11b mRNA | Liver | One-way ANOVA | η² | 0.06 | Moderate |
| Fig. 4H | CD86 mRNA | Liver | One-way ANOVA | η² | 0.15 | Large |
| Fig. 4I | CD206 mRNA | Liver | One-way ANOVA | η² | 0.88 | Large |
| Fig. 4K | LPL protein expression | Liver | One-way ANOVA | η² | 0.08 | Moderate |
| Fig. 4L | PGC1α protein expression | Liver | One-way ANOVA | η² | 0.31 | Large |

Legend: Effect sizes (η²) were calculated from one-way ANOVA models applied to hepatic gene and protein expression analyses. Effect size magnitude was interpreted using conventional thresholds for eta-squared: small (<0.06), moderate (0.06–0.13), and large (≥0.14), providing an estimate of the proportion of variance explained by experimental condition. This analysis highlights selective modulation of hepatic metabolic and immunometabolic pathways, with large effect sizes observed for markers related to fatty acid oxidation and lipid handling (FABP4, HSL, CPT1α, PGC-1α) and for CD206, while classical lipogenic and inflammatory markers exhibited small or small–moderate effects.

**Table S9: Global effect size estimates for PVAT outcomes**

| **Figure / Panel** | **Outcome** | **Tissue** | **Test** | **Effect size metric** | **Effect size value** | **Interpretation** |
| --- | --- | --- | --- | --- | --- | --- |
| Fig. 5A | PVAT mass | PVAT | One-way ANOVA | η² | 0.77 | Large |
| Fig. 5C | Adipocyte area | PVAT | One-way ANOVA | η² | 0.029 | Small |
| Fig. 5D | Adipocyte number | PVAT | One-way ANOVA | η² | 0.79 | Large |
| Fig. 5E | Leptin | PVAT supernatants | Kruskal–Wallis | ε² | 0.27 | Large |
| Fig. 5F | TNF-α | PVAT supernatants | One-way ANOVA | η² | 0.21 | Large |
| Fig. 5G | MCP1 | PVAT supernatants | One-way ANOVA | η² | 0.37 | Large |
| Fig. 5H | IL-10 | PVAT supernatants | Kruskal–Wallis | ε² | 0.42 | Large |
| Fig. 5I | F4/80 mRNA | PVAT | One-way ANOVA | η² | 0.51 | Large |
| Fig. 5J | CD80 mRNA | PVAT | One-way ANOVA | η² | 0.28 | Large |
| Fig. 5K | CD86 mRNA | PVAT | One-way ANOVA | η² | 0.41 | Large |
| Fig. 5L | CD163 mRNA | PVAT | One-way ANOVA | η² | 0.25 | Large |
| Fig. 5M | CD301 mRNA | PVAT | One-way ANOVA | η² | 0.18 | Large |
| Fig. 5N | CD206 mRNA | PVAT | One-way ANOVA | η² | 0.72 | Large |
| Fig. 5O | iNOS mRNA | PVAT | One-way ANOVA | η² | 0.9 | Large |
| Fig. 5P | Arginase mRNA | PVAT | One-way ANOVA | η² | 0.34 | Large |
| Fig. 6D | pAMPK/AMPK | PVAT | One-way ANOVA | η² | 0.58 | Large |
| Fig. 6E | peNOS/eNOS | PVAT | Krushal-Wallis | ε² | 0.40 | Large |

Legend: Effect sizes were classified as small (η² < 0.06), moderate (η² = 0.06–0.13) or large (η² ≥ 0.14). For non-parametric analyses, epsilon-squared (ε²) values ≥ 0.26 were considered large.

**Table S10: Effect size analysis of vascular reactivity and cardiovascular parameters**

| **Figure / Panel** | **Outcome** | **Statistical model** | **Factor** | **Effect size metric** | **Effect size value** | **Interpretation** |
| --- | --- | --- | --- | --- | --- | --- |
| Fig. 6A | Vasorelaxation | Two-way ANOVA | Agonist (row) | η² | 0.47 | Large |
| Fig. 6A | Vasorelaxation | Two-way ANOVA | Group (column) | η² | 0.12 | Moderate |
| Fig. 6A | Vasorelaxation | Two-way ANOVA | Interaction | η² | 0.04 | Small |
| Fig. 6B | Vasoconstriction | Two-way ANOVA | Agonist (row) | η² | 0.60 | Large |
| Fig. 6B | Vasoconstriction | Two-way ANOVA | Group (column) | η² | 0.06 | Moderate |
| Fig. 6B | Vasoconstriction | Two-way ANOVA | Interaction | η² | 0.04 | Small |
| Suppl. Fig. S1.A | Systolic blood pressure | Two-way RM ANOVA | Time | η² | 0.15 | Large |
| Suppl. Fig. S1.A | Systolic blood pressure | Two-way RM ANOVA | Group | η² | 0.06 | Moderate |
| Suppl. Fig. S1.A | Systolic blood pressure | Two-way RM ANOVA | Time x Group | η² | 0.06 | Moderate |
| Suppl. Fig. S1.C | Diastolic blood pressure | Two-way RM ANOVA | Time | η² | 0.11 | Moderate |
| Suppl. Fig. S1.C | Diastolic blood pressure | Two-way RM ANOVA | Group | η² | 0.02 | Small |
| Suppl. Fig. S1.C | Diastolic blood pressure | Two-way RM ANOVA | Time x Group | η² | 0.10 | Moderate |
| Suppl. Fig. S1.E | Heart rate | Two-way RM ANOVA | Time | η² | 0.04 | Small |
| Suppl. Fig. S1.E | Heart rate | Two-way RM ANOVA | Group | η² | 0.13 | Moderate |
| Suppl. Fig. S1.E | Heart rate | Two-way RM ANOVA | Time x Group | η² | 0.18 | Large |

Legend: Effect sizes (η²) were calculated from two-way ANOVA or two-way repeated-measures (RM) ANOVA models used for the analysis of vascular reactivity (vasorelaxation and vasoconstriction), arterial blood pressure, and heart rate. For vascular assays, effect sizes are reported for the main effects of agonist concentration (row factor), experimental group (column factor), and their interaction. For cardiovascular parameters, effect sizes correspond to the main effects of time, experimental group, and time × group interaction. Interpretation of effect size magnitude followed conventional benchmarks for small (η² < 0.06), moderate (η² = 0.06–0.13) or large (η² ≥ 0.14).

**Supplemental figure S1: Chia oil supplementation does not alter systemic blood pressure or heart rate in obese mice.**


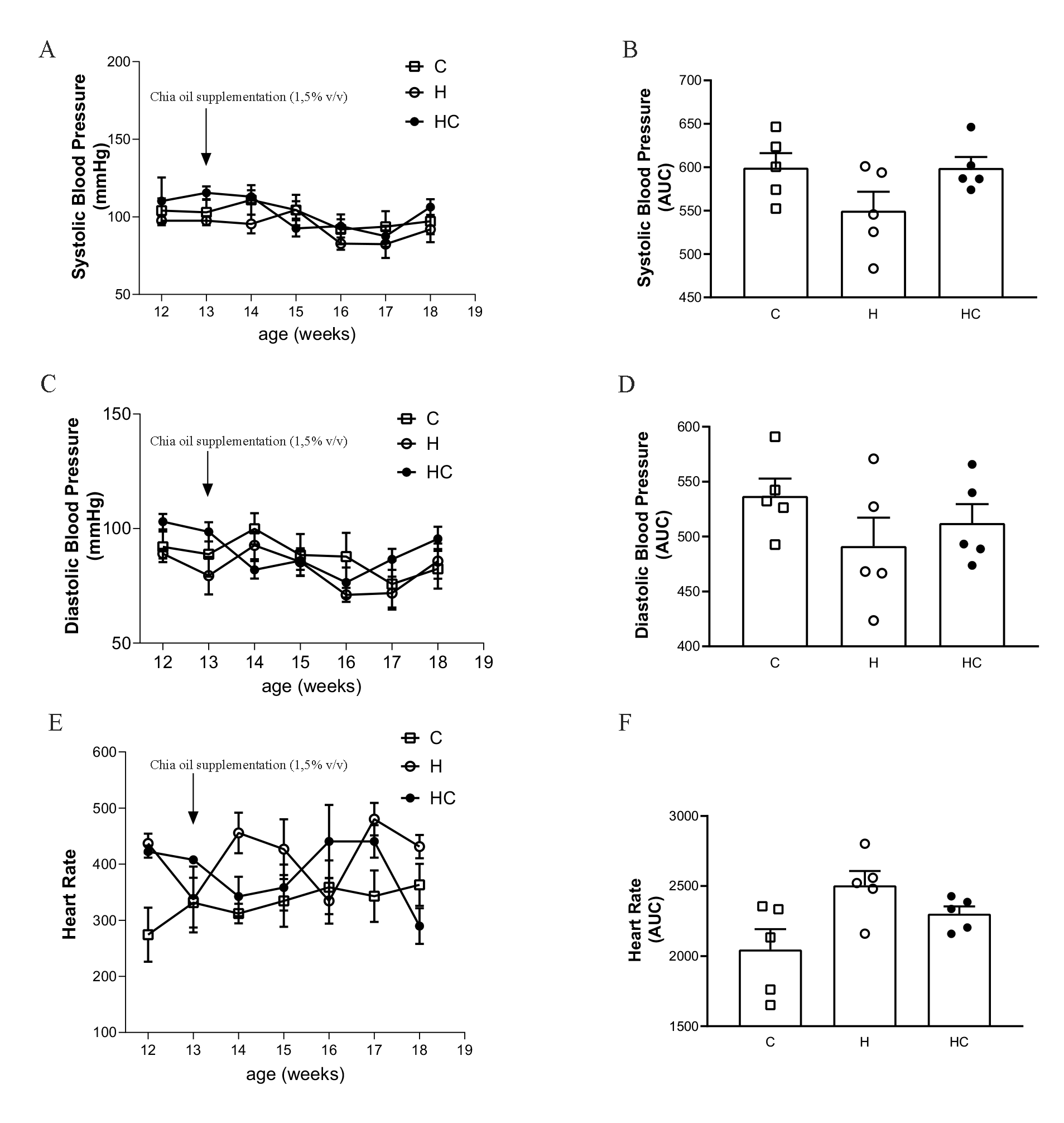


Legend: Chia oil supplementation does not alter systemic blood pressure or heart rate in obese mice. (A) Systolic blood pressure curve and (B) its area under the curve (AUC). (C) Diastolic blood pressure curve and (D) AUC. (E) Heart rate curve and (F) AUC. Measurements were obtained weekly via non-invasive tail-cuff plethysmography throughout the dietary intervention period. Data are presented as mean ± S.E.M. (one-way ANOVA followed by Tukey’s post hoc test or Kruskal-Wallis with Dunn’s test, as appropriate). n = 5 animals/group. C = Chow; H = High-fat diet; HC = High-fat diet + chia oil.
